# Enhanced near infrared spectral data to improve prediction accuracy in determining quality parameters of intact mango

**DOI:** 10.1016/j.dib.2020.105571

**Published:** 2020-04-21

**Authors:** Rita Hayati, Agus Arip Munawar, F. Fachruddin

**Affiliations:** a Department of Agro-technology, Syiah Kuala University, Banda Aceh, Indonesia; b Department of Agricultural Engineering, Syiah Kuala University, Banda Aceh, Indonesia; c Agricultural Mechanization Research Centre, Syiah Kuala University, Banda Aceh, Indonesia

**Keywords:** Datasets, Enhancement, Spectra, NIRS, Mango

## Abstract

Presented manuscript aimed to describes enhanced near infrared spectral dataset used to improve prediction performances of near infrared models in determining quality parameters of intact mango fruits. The two mentioned quality parameters are total acidity (TA) and vitamin C which corresponds to main inner attributes of fruits. Near infrared (NIR) spectra data were acquired and recorded as absorbance spectral data in wavelength range from 1000 to 2500 nm. These data were then enhanced by means of several algorithms like multiplicative scatter correction (MSC), baseline linear correction (BLC) and combination of them (MSC+BLC). Prediction models, used to determine TA and vitamin C were established using most common approach: partial least square regression (PLS) based on raw and enhanced spectral data respectively. Prediction performances can be evaluated based on prediction accuracy and robustness, by looking statistical indicators presented as coefficient of determination (R^2^) and correlation (r), root mean square error (RMSE) and residual predictive deviation (RPD). Enhanced NIR spectral dataset can be employed as a rapid, effective and non-destructive method to determine inner quality parameters of intact fruits.

Specifications Table

**Table:** 

Subject	Agricultural and Biological Sciences Horticulture
Specific subject area	Near infrared spectroscopy application in agriculture
Type of data	Table Graph Spectroscopic data
How data were acquired	Near infrared spectral data of intact mango fruits were acquired using a benchtop infrared instrument (Thermo Nicolet Antaris II TM) in the wavelength range from 1000 to 2500 nm with 0.2 nm resolution windows. The light source of halogen lamp irradiated fruit samples through a quartz window with 1 cm of diameter. Intact fruit was placed manually upon sample holder embedded in the top of the NIR instrument. Background spectra correction was carried out automatically once every 10 sample acquisitions. Raw or original spectral data were collected and recorded as absorbance spectrum in the presence of energies from 4000 to 10 000 cm^−1^ and then converted onto wavelength (1000 - 2500 nm) for a total of 58 intact mango samples (var. *Kent*). Each spectra data consisted of 1557 wavelength variables as an average of 32 successive spectra data acquisition. Moreover, enhanced spectra datasets were acquired by transforming and correcting raw spectra data by means of specified algorithms: multiplicative scatter correction (MSC), baseline linear correction (BLC) and combination of MSC and BLC. These raw and enhanced spectral data were then used to construct prediction models for inner quality parameters determination.
Data format	Raw Analysed Enhanced Presented as *.unsb* and *.xlsx* file formats
Parameters for data collection	Enhanced and original raw spectral datasets of intact mango fruits were used to predict two main inner quality attributes presented as total acidity (TA) and vitamin C.
Description of data collection	Predicted value of total acidity and vitamin C of mango fruits were collected by constructing and establishing prediction models based on spectral datasets. Those models were developed using the most commonly used approach: partial least square regression (PLS) followed with cross validation to avoid over fitting models. Yet, prediction models can also be constructed using other regression approaches like principal component regression (PCR), stepwise and backward linear regression or even using non-linear regression approach. Spectral data were regressed with actual TA and vitamin C, obtained from standard laboratory measurements. Predicted value of TA and vitamin C were then compared with the actual measured TA and vitamin C to evaluate models performances.
Data source location	Near infrared spectra dataset of intact mango samples and inner quality parameters data (Ta and vitamin C) were collected at the Faculty of Agriculture, Georg-August University of Goettingen, Germany.
Data accessibility	Dataset are available on this article and can be found in Mendeley repository data: https://data.mendeley.com/datasets/ph57ynng46/1 or http://dx.doi.org/10.17632/ph57ynng46.1

## Value of the Data

• Enhanced spectral dataset based on near infrared spectroscopy can be benefited for horticulture communities and industries especially for rapid quality inspection and evaluation.
• Datasets can be reused and remodelled to predict other quality parameters such as fibre and soluble solids content (SSC) of intact mango fruits by means of different regression approaches like support vector regression and artificial neural networks.
• Enhanced spectral data coupled with proper regression approach can be applied to predict inner quality parameters of intact fruits accurately.

## 1 Data

Spectral data based on near infrared spectroscopy has become more attractive to be analysed and modelled due to its importance in many field of applications including agriculture. These data mainly can be used to determine inner quality parameters of agricultural products such as fruits, vegetables and their derivative products. In food and agriculture communities and practices, quality of their products is important issue. Consumers are gradually looking for agricultural products with good quality since it is related to health aspect of human body. They need to be ensured those agricultural products are in good condition physically and chemically. In order to determine fruits and other agricultural products quality, several methods like titrations and solvent extractions were widely employed. However, most of those methods are based on complicated and time consuming methods which also destructive in nature [1]. Therefore, rapid, effective and non-destructive methods are required as an alternative in determining inner quality parameters of agricultural products.

**Fig. 1:**
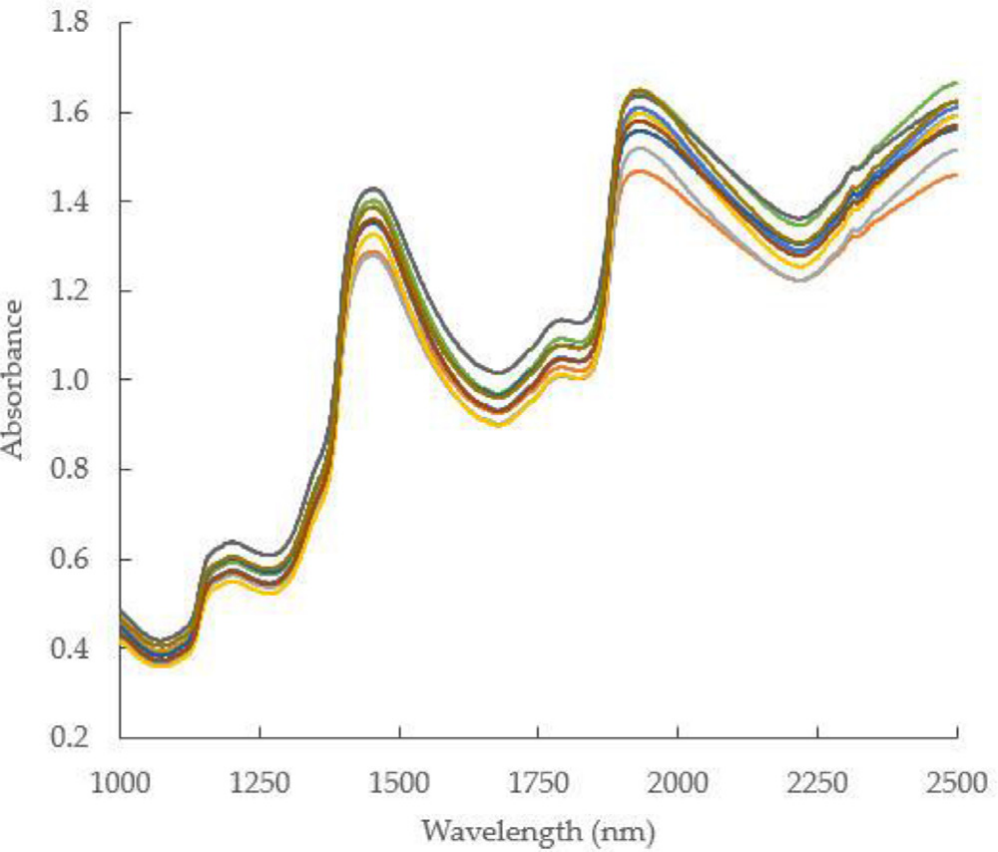
near infrared absorbance spectra of intact mango samples

Near Infrared spectroscopy (NIRS) has been widely proposed and applied as an alternative method for determining inner quality parameters of foods and agricultural products, including fruits. Near infrared spectra data can be presented as reflectance, absorbance or transmittance spectral data [2,3]. This technique has rapidly grown and developed into a fast, effective and robust analytical method for many fields including in agriculture. The NIRS works based on the interaction between biological object and electromagnetic radiation in the near infrared (NIR) region [4,5]. In agriculture application, the biological object is irradiated with light, while the radiation penetrates into the object, its spectral characteristics changes through wavelength dependent scattering and absorption process. The contribution of each reaction depends on the inner quality attributes, chemical and physical properties, and cell structure of the object [6]. Spectra data presented as absorbance in the wavelength range from 1000 to 2500 nm for intact mango samples are shown in Fig. 1. Absorbance spectra data for fruit samples absorbed energies which corresponds to chemical or inner quality parameters such as vitamin C, sugar contents, acidity, and fibre content.

**Fig. 2:**
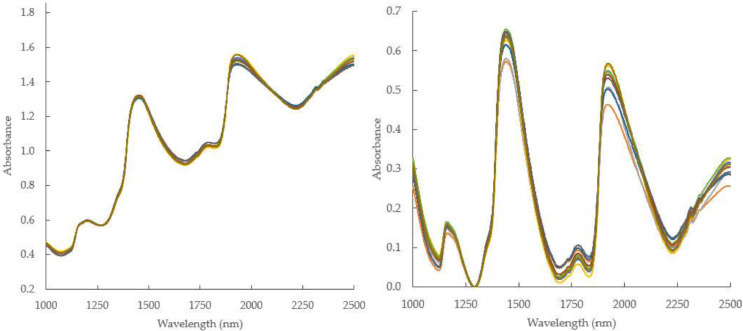
Enhanced spectra data of intact mango samples after MSC (a) and BLC (b) algorithms.

As shown in Fig. 1, inner quality properties like vitamin C, total acidity (TA), and other information related to mango quality parameters, were buried in spectra data. Spectra feature related to the absorption molecules of O-H, C-H, C-H-O and can be revealed through modelling by means of regression techniques and other specific mathematical approaches. Spectra data were used in combination with measured actual inner quality parameters to develop prediction models used to determine those inner attributes in future samples. Spectra datasets can also be corrected and enhanced to improve prediction accuracy and robustness. There are many methods that can be employed to enhance spectra data, and among of them are multiplicative scatter correction (MSC) and baseline linear correction (BLC) (see Fig. 2).

To determine inner quality parameters of intact mango samples, spectra data either as raw spectra or enhanced spectra data must be modelled through a process called calibration using several regression approaches. The two most common methods in NIR calibration are principal component regression (PCR) and partial least square regression (PLS) [7,8]. The PCR is a two-step procedure started with decomposing spectra data of mango fruits as X-variables using principal component analysis (PCA), and then continued with fitting a multiple linear regression model, using a small number of principal components (PCs) or latent variables (LVs) instead of full original variables as predictors which of course hundreds or even thousand numbers of variables [9,10]. In this datasets, each reading spectrum consisted a total of 1557 numbers (obtained from 1000 to 2500 nm with the increment approximately 0.2 nm) of full original variables. It is obvious that if we used all original spectrum instead of small number of PCs or LVs, it would be ineffective and may cause model with lack of accuracy and overall performances.

On the other hand, the PLS is an approach with somehow close likely to PCR one. The main difference of the PLS method to PCR is that both spectra data of intact mango samples (X-variables) and their actual data of inner quality parameters like TA and vitamin C data (Y-variables) (see Table 1), are projected onto new spaces. In PLS approach, an orthogonal basis of latent variables or factors is constructed one by one in such a way that they are oriented along the directions of maximal covariance between the spectral matrix and the response vector [11]. Partial least square algorithm ensured that all latent variables are ordered according to their relevance for predicting total acidity (TA) and vitamin C or other quality properties.

**Table 1:** Descriptive statistics data of actual measured total acidity and vitamin C of mango fruits.

Statistical parameters	Total Acidity (TA)	Vitamin C
Mean	495.48	32.21
Max	772.77	35.66
Min	189.72	28.93
Range	583.05	6.73
Std. Deviation	131.07	1.31
Variance	17179.68	1.71
RMS	512.24	32.23
Skewness	-0.16	0.14
Kurtosis	-0.17	1.12
Median	490.81	32.13
Q1	413.21	31.59
Q3	582.93	32.74

Q1: first quartile, Q3: third quartile.

**Fig. 3:**
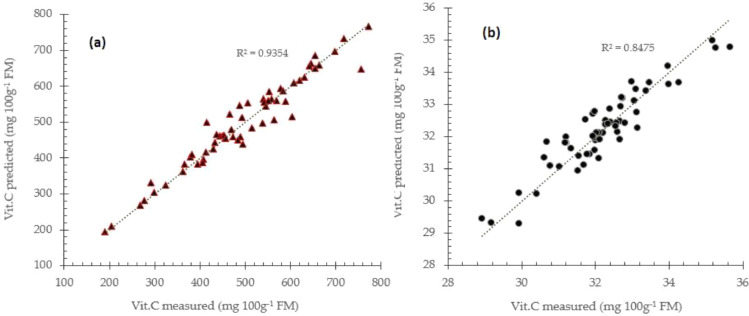
Prediction performance for total acidity (a) and vitamin C (b) prediction using raw spectra data with partial least square regression approach.

The main advantage of PCR and PLS models compared to common multiple linear regression (MLR) is that the X-variables which corresponds to principal components or latent variables are uncorrelated, and the noise is filtered. Further, usually a small number of principal components and factors are preferable for the effectiveness of the prediction models [12], [13], [14]. The number of latent variables or principal components required to construct prediction models is also taken into a consideration to avoid model overfitting. Prediction performance using raw spectra data for TA and vitamin C prediction using partial least square regression approach is presented in Fig. 3 respectively.

The partial least square regression (PLS) attempted to find best correlation between near infrared spectra data and inner quality parameters of intact mango like total acidity and vitamin C. To date, the PLS method is still widely applied NIRS practice and application. The PLS approach seems to be fitted in many NIRS applications including in agriculture. In fact, near infrared spectra data of fruits and vegetables is essentially composed of a large set of overtones and combination bands. Spectra data are mainly influenced by wavelength dependant scattering effects, tissue heterogeneities of agricultural object, instrumental noise, ambient effects and other source of variability [15]. Therefore, spectra data correction and enhancement sometime are genuinely required to eliminate or minimize caused noises.

## 2 Experimental design, materials, and methods

### 2.1 Materials and instrument setup

Datasets for near infrared spectra data and actual measured TA and vitamin C were obtained for a total of 58 mango fruit samples (cv. Kent) selected from two different origins: Brazil and Spain. All mango samples were obtained and purchased in local market in Goettingen, Gernany. Mango fruits were stored for 10 days at ambient temperature of 25°C and measured every two days in order to have samples with varied maturity, since mango is categorized as climacteric fruits.

Near infrared spectroscopy instrument (Thermo Nicolet Antaris II) was used as main instrument for acquiring and recording spectra data. It was controlled using integrated software namely Thermo Integration® and Thermo Operation®. The workflow has been developed to run specified tasks for spectra data acquisition from which high resolution measurement with integrating sphere was chosen as a basic for obtaining spectra data. Sample labelling was also required every single measurement to distinct all 58 samples [2]. Background spectra correction was carried out every 10 samples acquisition automatically. Spectra datasets were set to be recorded and saved in local computer as *.spa*, and *.csv* extension file formats respectively. Moreover, standard laboratory method has been also prepared for actual total acidity (TA) and vitamin C measurements. Automatic titration equipment was used to determine actual TA, while standard titration method was applied to determine vitamin C of mango samples. On the other hand, the centrifuge was also used to obtain clarified sample juice and separate suspended solids of mango fruit samples.

### 2.2 Spectra data acquisition

Near infrared spectra data for intact mango fruits were acquired in wavelength range from 1000 to 2500 nm or in wavenumber 4000 to 10 000 cm^−1^ and recorded as absorbance spectra data. Each sample was placed centrally upon fruit holder at the top of NIRS instrument. It placed exactly to the incoming light hole with 1 cm of diameter to ensure direct contact and minimize noises. Spectra data were acquired as a successive average of 64 scans per sample [16]. Background spectra correction was carried out automatically once every 10 sample acquisitions to ensure reliable spectra data [17,18].

### 2.3 Total acidity and vitamin C data measurement

Once after spectra data acquisitions were completed, actual total acidity and vitamin C of mango fruits were directly measured. The vitamin C content was performed firstly since this parameter is easily evaporated after pulp slicing. Five grams of fruit pulp sample was macerated, mixed and homogenized with a total of 20 ml meta-phosphoric acid into a beaker to prevent rapid oxidation. Distilled water was then added until 50 ml of volume was reached. The vitamin C content was quantified based on its reaction with the *Dichlorophenolindophenol* as an indicator in titration method from which colour change from colourless to light red at the end of titration [11]. Vitamin C content of fruit samples were expressed in mg.100g^−1^ fresh mass (FM). Moreover, to obtain total acidity (TA) data of mango fruit, another juices from 20 grams of fruit pulp sample and maximum 100 ml distilled water. In order to obtain clarified sample juice and separate suspended solids, the centrifuge (20-26°C, 10 000 g) was applied for about 10 minutes. A 10 mL filtered juice was taken and transferred into a beaker glass and titrated in automatic titrator equipped with 0.1 N NaOH to an end point of pH 8.1. The total acidity data can be read from the equipment display and it expressed as mg.100g-1 FM.

### 2.4 Spectral data enhancements

As mentioned previously, spectra data can be corrected and enhanced to improve prediction accuracy and robustness. Obtained spectra data sometimes contained noises and irrelevant background information due to internal and external factors such as light scattering, over-heated sensors and others. These noises can interfere quality properties information required to be revealed from spectra data. Therefore, it is recommended to correct and enhance spectra data prior to constructing prediction models. There are many spectra enhancement methods widely available with different algorithms such as derivation, normalization and transformation.

Among those spectra enhancement methods, multiplicative scatter correction (MSC) is one of the most common and popular correction methods for NIRS users. The MSC is used to compensate for additive and multiplicative effects in the spectral data caused by physical effects. It also proven to be effective and robust spectra correction method among others. This method attempted to remove the effects of scattering by linearizing each spectrum to an *ideal* spectrum of the spectra data which is corresponds to the average spectrum. Moreover, baseline linear correction (BLC) is also commonly used for some users due to its ability to remove noises especially at the end of NIR wavelength at around 2300 – 2300 nm. Spectra correction methods can also be combined among those methods to generate more accurate and robust prediction results as shown in Table 2 and 3 respectively.

**Table 2:** Comparisons among different spectra data in determining total acidity of mango fruits using PLSR approach with optimum number of LVs 6.

Spectra data	Statistical indicators			
	R^2^	r	RMSE	RPD
Raw	0.935	0.967	24.143	5.429
MSC	0.959	0.979	21.624	6.061
BLC	0.947	0.973	22.407	5.850
MSC+BLC	0.976	0.988	19.351	6.773

BLC: baseline linear correction, MSC: multiplicative scatter correction, R^2^: coefficient of determination, r: correlation coefficient, RMSE: the root mean square error, RPD: residual predictive deviation.

**Table 3:** Comparisons among different spectra data in determining vitamin C of mango fruits using PLSR approach with optimum number of LVs 6

Spectra data	Statistical indicators			
	R^2^	r	RMSE	RPD
Raw	0.847	0.920	0.483	2.712
MSC	0.875	0.935	0.425	3.082
BLC	0.860	0.927	0.448	2.924
MSC+BLC	0.958	0.979	0.417	3.141

BLC: baseline linear correction, MSC: multiplicative scatter correction, R^2^: coefficient of determination, r: correlation coefficient, RMSE: the root mean square error, RPD: residual predictive deviation.

In addition, spectra data can be observed firstly to detect some odd spectra data which are known as outliers. The most common method used to detect outlier data is a combination of principal component analysis (PCA) and Hotelling t^2^ ellipse. Outliers are spectra data from fruit samples that considerably different from the other majority of remaining samples. They need to be treated carefully because they can influence whole prediction performance of NIRS prediction models. Dataset need to be improved by removing outliers and enhancing spectral data to achieve more accurate and robust prediction performances.

### 2.5 Prediction models

**Fig. 4:**
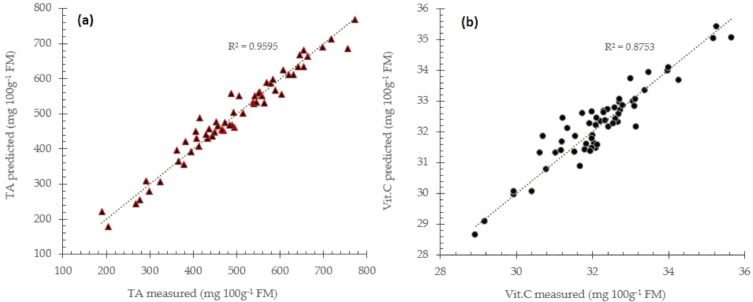
Prediction performance for total acidity (a) and vitamin C (b) prediction using MSC enhanced spectra data with partial least square regression approach.

**Fig. 5:**
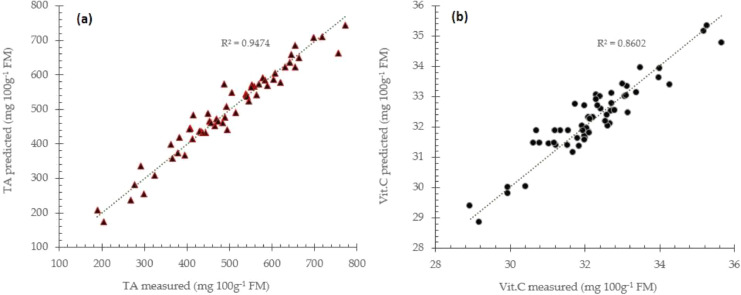
Prediction performance for total acidity (a) and vitamin C (b) prediction using BLC enhanced spectra data with partial least square regression approach.

To obtain information about inner quality parameters of mango fruits which buried in the spectra data, calibration must be carried out to construct prediction models. These models were developed by means of specific multivariate analysis from which partial least squares regression (PLS) was applied. Another regression approach like principal component regression (PCR) or stepwise and backward linear regression can also be used in calibration to predict desired quality attributes of intact fruits [19]. The methods adopted regression either as linear or non-linear regression approach from which spectra data (X-data) and actual measured quality parameters (Y-data) were regressed. Sample dataset used in calibration must be representative from which all expected sources of variability must be considered. To avoid overfitting and obtain reliable prediction models, cross validations can be performed during calibration. There are three most popular cross validation methods namely: k-fold cross validation, leverage validation and test matrix. Enhanced spectra generally can achieve better prediction accuracy compared to raw spectra data (see Figs. 4 and 5).

The performance of the prediction models were evaluated by using the following statistical indicators: the coefficient of determination (R^2^) which essentially represents the proportion of explained variance of the response in the dataset, coefficient of correlation (r) between predicted and measured quality attributes (TA and vitamin C), prediction error which is defined as the root mean square error (RMSE), and the residual predictive deviation (RPD) defined as the ratio between standard deviation of the actual measured quality parameters and the root mean square error (RMSE), standard error of cross validation or prediction performance (RMSECV or RMSEP). The higher the value of RPD, the greater probability of the model to predict TA, vitamin C and other chemical constituent in samples set accurately.

**Fig. 6:**
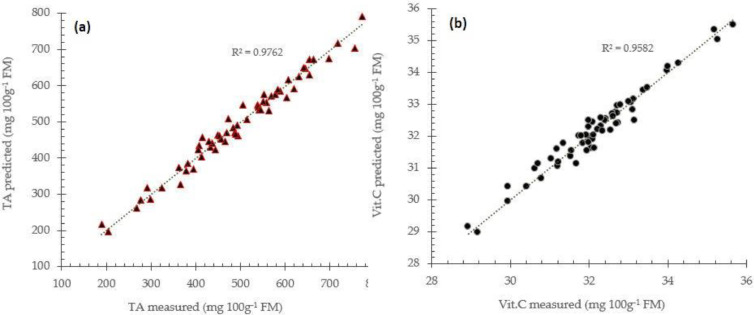
Prediction performance for total acidity (a) and vitamin C (b) prediction using a combination MSC+BLC enhanced spectra data with partial least square regression approach.

The RPD value between 1.5 and 2 means that the model can predict with sufficient performance and need improvement in the models., Meanwhile, RPD value between 2 and 2.5 indicates that coarse quantitative predictions are possible, and RPD between 2.5 and 3 or RPD above 3 corresponds to good and excellent prediction performances respectively. These values give the average uncertainty that can be expected for predictions of future samples. The RPD value can increase more and improve when the models are constructed using a combination of enhanced spectra data like MSC+BLC (see Fig. 6).

Combination can be carried out for more than two spectra enhancement methods. Also, the order can also be changed accordingly. However, the knowledge on how each spectra enhancement works is essential to be recognized, so that combined spectra enhancement approaches can significantly affected to the overall prediction performances of NIRS models.

Furthermore, beside using linear regression methods like PCR, PLS or stepwise and backward MLR, prediction models can be developed by means of non-linear regression approaches such support vector machine regression (SVR) and artificial neural networks (ANN) regression based [11],. The SVR and ANN approaches are more likely flexible calibration methods since they can handle both linear and non-linear relationship between near infrared spectra data and desired quality parameters of mango fruits.

## Appendix Supplementary materials

Image, application 1
